# Long-term efficacy of interventions for actinic keratosis: protocol for a systematic review and network meta-analysis

**DOI:** 10.1186/s13643-019-1156-8

**Published:** 2019-10-11

**Authors:** Theresa Steeb, Markus V. Heppt, Lars Becker, Christoph Kohl, Lars E. French, Carola Berking

**Affiliations:** Department of Dermatology and Allergy, University Hospital of LMU, Frauenlobstr. 9-11, 80337, Munich, Germany

**Keywords:** Skin neoplasms, actinic keratosis, actinic keratoses, Network meta-analysis, Cancer of skin, Skin cancer

## Abstract

**Background:**

Actinic keratoses (AK) are common precancerous lesions of the skin due to cumulative sun exposure. A variety of interventions are available for the treatment; however, the majority of randomised controlled trials (RCTs) and meta-analyses focus on short-term efficacy outcomes. This network meta-analysis aims to investigate the long-term (> 12 months) efficacy of interventions for AK.

**Methods:**

To identify relevant studies, we will perform a systematic literature research in MEDLINE, Embase, and CENTRAL and hand-search pertinent trial registers. Two authors will independently screen titles and abstracts for eligibility. We will include RCTs with an inter-individual (parallel arm) design. The study population includes patients with a clinical or histopathologic diagnosis of AK. Eligibility will be restricted to the following interventions: surgical approaches, cryosurgery, ablative laser treatment, topical drug treatment with 5-fluorouracil, imiquimod, ingenol mebutate, diclofenac, or photodynamic therapy. As outcomes, we will consider the following endpoints: (1) the participant complete clearance rate, (2) the participant partial clearance rate, (3) the lesion-specific clearance, (4) the mean lesion reduction per patient, and (5) the number of withdrawals due to adverse events after at least 12 months after the end of treatment. Monotherapy or placebo will serve as a comparison. Estimates of effects from individual studies will be pooled using a random-effects model. Heterogeneity will be evaluated based on *I*^2^ and chi-square test. The risk of bias will be estimated with the Cochrane Risk of Bias Tool by two review authors independently. The quality of evidence of the outcomes will be assessed with the GRADE approach. A network meta-analysis will be performed to combine direct and indirect evidence from the included RCTs.

**Discussion:**

The potential of interventions to achieve a sustained clearance of AK has not been assessed to date. To investigate the long-term efficacy of interventions is important as the natural disease course is highly variable and relapses occur frequently even after initial lesion clearance. This review will help to set a framework for clinical decision making in patients with AK.

**Systematic review registration:**

CRD42018095903 (PROSPERO)

## Background

Actinic keratoses (AK) are common precancerous lesions of the skin as a consequence of lifelong exposure to ultraviolet (UV) radiation [1, 2]. They belong to the most common skin lesions with a prevalence of up to 60% in Caucasians over the age of 60 years and may progress into invasive cutaneous squamous cell carcinoma (cSCC), although the risk is presumably low for single lesions [3]. However, if multiple AK are present and accompanied by signs of chronic actinic damage or field cancerization, the risk for malignant progression increases rapidly [4, 5]. As it is difficult to predict if a lesion will become an invasive cSCC, international guidelines recommend early consequent treatment of AK [6, 7]. A further motivation of treatment is to improve the cosmetic appearance as AK usually present as scaly erythematous or brownish lesions on the face or scalp.

A variety of interventions are available for the treatment of AK in clinical practice including topical drugs and ablative modalities. A myriad of studies has been published and showed that most interventions are superior to placebo in terms of lesion clearance and improving the cosmetic image. However, the majority of randomised controlled trials (RCTs) and meta-analyses focus on short-term outcomes that are evaluated by an investigator after few months [8–12], although AK are considered to be a chronic disease. Besides, most treatment modalities were investigated versus placebo and head-to-head comparisons are widely lacking, limiting the possibility to compare distinct active treatments. Additionally, until now, no evidence-based recommendation regarding the long-term efficacy of interventions for AK exists [6]. Hence, the aim of this study is to perform a systematic review with a network meta-analysis to explore the current knowledge on the long-term efficacy of interventions for patients with AK.

## Methods/design

### Protocol and registration

The protocol for this review was defined a priori and registered online in the PROSPERO international prospective register of systematic reviews (CRD42018095903). This protocol was conducted in accordance with the Preferred Reporting Items for Systematic Reviews and Meta-Analyses (PRISMA) as well as its extension for network meta-analyses (PRISMA-NMA) for protocols to BioMed Central journals (PRISMA-P) [13–16]. The checklist can be obtained from the Additional file 1.

### Objectives

This systematic review and meta-analysis aims to investigate the long-term (> 12 months) efficacy of interventions for AK.

### Types of studies

We will only include RCTs in which study participants (inter-individual, parallel-arm trials) are investigated. Dose-finding studies, pseudo-randomised trials, cross-over trials, observational studies, retrospective studies, and case series will be excluded. RCTs with an intra-individual design (i.e., treatment applied to opposite sides of the face or scalp) will not be considered due to statistical issues which may arise from pooling inter- and intra-individual trials [17]. Outcomes will be assessed 12 months or later after the end of treatment to allow for a powerful conclusion of the long-term efficacy. Thus, the observation time of the included studies has to be at least 12 months for inclusion. In the interim (between the end of treatment and outcome assessment) no additional intervention or repetition of the same intervention will be allowed.

### Types of participants

We will include adult patients (≥ 18 years of age) with a clinical or histopathological diagnosis of AK. Studies investigating immunosuppressed patients or organ transplant recipients will not be included as it is likely that they have a treatment response distinct from immunocompetent populations [8]. Furthermore, there is evidence that the natural course of AK is less favourable in immunocompromised individuals with a higher likelihood for conversion to invasive cSCC and lower rates of spontaneous resolution [18].

### Types of interventions

This study will be performed from a European perspective. Hence, we will focus on standard treatments which are available, approved, and regularly used in the clinical practice in European countries. We will include the following types of intervention: surgical approaches (such as excisional biopsy or shave excision), cryosurgery, cryopeeling, ablative lasers (such as erbium:YAG or carbon dioxide laser), ingenol mebutate 0.015% or 0.05% gel, imiquimod 3.75% or 5% cream, 5-fluorouracil (5-FU) 0.5% or 5% cream, 5-FU 0.5% plus salicylic acid 10% in solution, 3% diclofenac in 2.5% hyaluronic acid, and photodynamic therapy with aminolevulinate or its ester methyl-aminolevulinate with illumination from light-emitting diodes or natural daylight. Monotherapy of the interventions mentioned above or placebo will serve as a comparison. Sequential or combination approaches will be excluded as the focus of this review lies on the efficacy of the individual treatment options.

### Types of outcome measures

We will address the following outcomes: (1) participant complete clearance, defined as the number of patients with 100% cleared lesions (dichotomous outcome), (2) participant partial clearance, defined as the number of patients who have at least 75% cleared lesions (dichotomous outcome), (3) the lesion-specific clearance rate, defined as the number of cleared lesions compared from baseline to assessment (dichotomous outcome), (4) the mean reduction of lesions from baseline (or percentage reduction) per patient (continuous outcome), and (5) safety, defined as the number of patients experiencing at least one treatment-related adverse event leading to withdrawal from the study (dichotomous outcome). The endpoints need to be reported at least 12 months after the end of treatment. If several time points are reported in a primary study, data from the last reported time point will be used for the analysis.

### Search methods for identification of studies

We will search the electronic databases MEDLINE, Embase (both via Ovid), and the Cochrane library CENTRAL to identify all relevant records. The search strategies are shown in the Additional file 2. Additionally, we will search the following trial registers for the keywords “actinic keratosis” or “actinic keratoses”: The metaRegister of Controlled Trials (ISRCTN registry www.controlled-trials.com), US National Institutes of Health Ongoing Trials Register (www.clinicaltrials.gov), Australian New Zealand Clinical Trials Registry (www.anzctr.org.au), World Health Organization International Clinical Trials Registry Platform (www.who.int/trialsearch/), EU Clinical Trials Register (www.clinicaltrialsregister.eu/). For ongoing trials and completed trials without data publication, principal investigators or trial sponsors will be contacted to obtain preliminary or unpublished data. Reference lists of included records will be screened as well.

### Selection of studies

Two authors (TS, LB) will independently screen titles and abstracts for eligibility that were identified in the electronic database searches. Trial registers will be hand-searched and assessed for eligibility by one author (LB). For records that are considered relevant according to title and abstract screening, full-text articles will be obtained, and inclusion and exclusion criteria will be applied. Whenever discrepancies arise, a resolution will be achieved by discussion with a third independent author (MVH). For screening purposes, records will be imported into the reference managing software Endnote X8 (Thomson Reuters).

### Data extraction and management

Information for each included study will be collected by two authors independently (TS, LB) to a specifically developed data extraction form using Microsoft Excel 2010. The data extraction form will be piloted. Information will be collected on study characteristics (author, year of publication, study design, number of arms, sample size, duration of follow-up), participant characteristics (age, sex, numbers of participants, and localization of AK), intervention and comparator details (sample size for each treatment arm, dosage and type of intervention, duration of treatment, withdrawals, and drop-outs), as well as on the pre-defined efficacy outcomes.

The adjusted analyses of treatment effects will be used, if available in the primary studies. Otherwise, we will include the unadjusted data as reported in the original RCTs. Data will be imported from the collection sheet as csv-file to the software R (version 3.5.1) by one author (MVH) and double-checked by a second person (TS) [19]. Reviewers will not be blinded to study author, institution, or journal.

### Assessment of risk of bias

Two authors will independently assess the studies’ risk of bias with the Cochrane Risk of Bias tool for RCTs using Review Manager 5.3 [20]. Discrepancies will be thoroughly discussed and resolved with the full texts and supplementary material. The following domains will be investigated: random sequence generation, allocation concealment, blinding (of participants, personnel, and outcome assessors), incomplete outcome data, and selective outcome reporting and other sources of bias for the RCTs. The items will be described as having ‘low’, ‘high’, or ‘unclear’ risk of bias (see Cochrane Handbook for Systematic Reviews of Interventions). The risk of bias assessment will be done both on the outcome and study level. In order to minimise the chance of influence of publication bias, we will perform a comprehensive search for eligible studies including trial registries. If at least 10 RCTs report outcomes for a specific comparison, we intend to assess publication bias by creating a funnel plot [21].

### Assessment of heterogeneity

Statistical, clinical, and methodological heterogeneity will be examined prior to conducting meta-analysis. We will estimate the magnitude of statistical heterogeneity using the restricted maximum likelihood and the Q-profile method to estimate the 95% CI for dichotomous outcomes [22]. For continuous outcomes, we will use the Friedman or Durbin test. The proportion of variability that is due to heterogeneity rather than sampling error will be assessed based on *I*^2^ and the statistical test chi-square. Overall, when significant heterogeneity will be found amongst comparable studies (*I*^2^ for heterogeneity > 75%), pooled estimates will not be provided. Instead, we will describe the results qualitatively, as suggested in the Cochrane Handbook for Systematic Reviews of Interventions (Chapter 9) [21]. Heterogeneity will be explored with subgroup and sensitivity analyses, as well as with meta-regression if applicable. We will use random-effects meta-regression models to quantify the difference between subgroups and test for statistically significant interactions amongst subgroups. Beyond statistical heterogeneity, clinical heterogeneity will be evaluated based on the comparability of the trials in terms of clinical issues, such as no major imbalances or differences in the included population or intervention. To address the influence of bias on the effect estimate, we will perform sensitivity analyses by repeating meta-analysis only with studies with an unclear or low risk for bias and additionally with small sample sizes (i.e., arms of less than 10 patients). Then, another sensitivity analysis will be conducted to examine whether effect estimates are influenced by the placebo effect investigated in the individual trials. Furthermore, major imbalances in the population that may influence the network such as the number and localization of lesions and the size of the affected area will be investigated. Regarding the intervention, off-label use is a potential factor that will be considered when performing this meta-analysis. Pooled effect estimates of studies which adjusted for confounders (e.g. low risk of bias) will be compared with pooled effect estimates of all studies (studies which adjusted and studies which did not adjust for confounders). If there are differences between these estimates, they will be considered in the results and discussion.

### Data synthesis

To assess treatment effects and effect sizes, dichotomous outcomes will be expressed as risk ratios with 95% confidence intervals. Each patient in the included study will be the unit of analysis for the participant complete and partial clearance, whereas single AK lesions will be the unit of analysis for the lesion-specific clearance rate. Where possible, we will calculate the data following the intention-to-treat principle. We will also contact principal investigators or study authors to obtain missing data. Imputation of missing data is not planned. If results are only reported graphically, we will graphically obtain the values, whenever applicable.

As we expect to include different comparisons of interventions, direct and indirect evidence will be combined with a network meta-analysis assuming independency and consistency of the included studies. We will use the frequentist approach using the R package “netmeta” and calculate a random-effects model [23]. Prior to analysis, data will be imported from the collection sheet in an arm-based format, enabling the inclusion of multi-arm trials [24]. Thereafter, they will be transformed from arm-based to contrast-based which is the necessary input format for the package “netmeta”. For the main analyses, we will choose placebo as the reference treatment. Treatments will be ranked with the P-score method using the function “netrank” [25]. Forest plots and two-dimensional graphical presentations of the network structure will be created with the same package in R [26].

Prior to performing the network meta-analysis, we will examine whether the distribution of the effect modifiers (number of lesions, localization of lesions, size of the affected area) is comparable across treatment comparisons [27]. The lack of transitivity in a network can create statistical disagreement between direct and indirect evidence, that is, inconsistency [28, 29]. Therefore, we plan to construct for each outcome a table of important patient characteristics and draw boxplots to visually inspect the distribution (for example, age) or percentages (for example, male/female) of factors we consider as potential modifiers of the treatment effect. Consistency between direct and indirect data will be examined locally, i.e., in certain paths of the network, using the loop-specific method [30, 31] and the separating indirect and direct evidence (SIDE) method [32]. Besides, consistency will also be examined globally, i.e., evaluating the network as a whole, using the design-by-treatment interaction model (DBT) [33]. If heterogeneity and/or inconsistency is observed, we will explore the possible sources, such as effect modifiers, as described previously.

### Rating the quality of evidence

The certainty of the evidence for the outcomes will be assessed using the GRADE approach for network meta-analyses in several steps [34–36]. First, the certainty of direct evidence will be assessed for risk of bias, inconsistency, indirectness, publication bias, and imprecision according to the GRADE principles. Second, indirect evidence will be rated based on the direct comparisons which inform the indirect evidence. We will focus on first-order loops, as we expect them to contribute the most information to the indirect estimates. First-order loops will be identified by a graphical illustration of the network using the function “netgraph” in the R package. The lowest confidence rating of the direct comparisons will constitute the rating of the indirect comparison and will further consider and be downgraded for intransitivity if applicable. Third, we will rate the network estimate based on the contributing direct and indirect evidence. If only either direct or indirect evidence exists, the network certainty will be assessed based on that estimate. If mixed evidence is present with different ratings, the highest quality between direct and indirect ratings will be used for the network estimate. In case we detect large incoherence or imprecision for a specific comparison, the network estimate will be downgraded. Based on these criteria, the certainty of the evidence for each comparison and outcome will be scored as high, moderate, low, or very low and will be presented separately for the direct, indirect, and network estimates.

### Funding

The project will be supported by a grant of the German Cancer Aid (grant no. 70112351) to CB. The funders did not have any role in developing this protocol.

### Discussion

The idea to systemically analyse the long-term efficacy of interventions for AK arises from the observation that AK are increasingly regarded as a chronic progressive condition due to lifelong exposure to UV [5]. Effective lesion clearance for a short term can be achieved by numerous ablative and topical interventions [6]. However, it is less clear if the efficacy can be sustained over a longer period of time without any further treatment. The question on the long-term results of interventions is gaining more and more importance as the disease course of patients with AK is hard to determine, ranging from direct malignant transition to invasive cSCC to spontaneous resolution without any active treatment [4, 37].

A scoping review of the publication landscape revealed that the paradigm shift in the understanding of the pathogenesis and significance of AK as chronic condition has not been translated into the design of major RCTs yet. The vast majority of trials published in the field reported efficacy endpoints on average 3–8 months after the end of treatment. Only a few studies provide results taken at later time points. Also, systematic reviews and meta-analysis of RCTs have so far fallen short of assessing the long-term efficacy of interventions [11, 38]. A previously published network meta-analysis of 10 established treatment options considered the participant complete clearance rates up to 12 weeks after the end of treatment [38]. Whilst the design of our endeavour is comparable to previous studies [11, 38], we will only include data reported at the earliest 12 months after treatment to specifically investigate the long-term results in our review.

To obtain a representative impression on the efficacy, we will employ four different efficacy endpoints. Two of them are participant-specific (participant complete clearance and participant partial clearance), and two are lesion-specific (lesion clearance rate and mean reduction of lesions from baseline). We believe that this redundancy is important in order to capture all possible results, as a core outcome set for a standardised reporting from RCTs for AK has not been defined yet. Thus, the inclusion of only one outcome bears the danger of missing important data and may lead to a systematic bias.

Potential threats to this project are a large number of different comparisons with lacking head-to-head comparisons. To still evaluate all treatment approaches against each other, we will perform both naïve comparator-specific pairwise analyses and a network meta-analysis. Furthermore, attrition of patients during the long observation time and selective reporting from patients who had received active treatments may pose a major challenge to this project. A long observation period may inflate the heterogeneity of the included studies. Nevertheless, we are confident that this study can overcome these challenges by the means described in this protocol and will help to set an evidence-based framework to guide treatment decisions in patients with AK.

## Supplementary information

**Additional file 1.** PRISMA-P 2015 checklist.

**Additional file 2.** Strategies for the database searches in MEDLINE, Embase, and CENTRAL.

## Data Availability

The datasets that will be created and analysed during the current study are available from the corresponding author on reasonable request.

## Abbreviations

AK: Actinic keratosis or actinic keratoses
CENTRAL: Cochrane Controlled Register of Trials
cSCC: Cutaneous squamous cell carcinoma
GRADE: Grading of Recommendations, Assessment, Development and Evaluation
RCT: Randomised controlled trial
PRISMA: Preferred Reporting Items for Systematic Reviews and Meta-Analyses
UV: Ultraviolet
5-FU: 5-Fluorouracil
