# Supplementary material for: Long-term efficacy of interventions for actinic keratosis: protocol for a systematic review and network meta-analysis
Source: Syst Rev. 2019 Oct 11;8:237. doi: 10.1186/s13643-019-1156-8 (PMC6788027; doi:10.1186/s13643-019-1156-8)
Supplement: Supplementary file 2 — Additional file 2. Strategies for the database searches in MEDLINE, Embase, and CENTRAL. [file 13643_2019_1156_MOESM2_ESM.docx]

**Additional file 2:** Strategies for the database searches in MEDLINE, EMBASE and CENTRAL.

(a) Search in MEDLINE via Ovid (June 13, 2018)

| **#** | **Searches** | **Results** |
| --- | --- | --- |
| 1 | actinic keratosis.mp. or exp Keratosis, Actinic/ | 2999 |
| 2 | solar keratosis.mp. | 218 |
| 3 | senile keratosis.mp. | 51 |
| 4 | field change.mp. | 311 |
| 5 | actinically damaged field.mp. | 4 |
| 6 | exp Precancerous Conditions/ or field-cancerized.mp. | 47616 |
| 7 | 1 or 2 or 3 or 4 or 5 or 6 | 49251 |
| 8 | excision.mp. | 138180 |
| 9 | exp Biopsy/ or shave.mp. | 261872 |
| 10 | curettage.mp. or exp CURETTAGE/ | 14943 |
| 11 | laser.mp. or exp Lasers/ | 272793 |
| 12 | cryotherapy.mp. or exp CRYOTHERAPY/ | 28649 |
| 13 | cryosurgery.mp. or CRYOSURGERY/ | 12966 |
| 14 | cryopeel*.mp. | 6 |
| 15 | fluorouracil.mp. or exp FLUOROURACIL/ | 54497 |
| 16 | fluorouracil derivative.mp. | 82 |
| 17 | efudix.mp. | 26 |
| 18 | actikerall.mp. | 5 |
| 19 | 5-FU.mp. | 17674 |
| 20 | exp Aminoquinolines/ or imiquimod.mp. | 24037 |
| 21 | aldara.mp. | 137 |
| 22 | zyclara.mp. | 4 |
| 23 | exp Diterpenes/ or ingenol mebutate.mp. | 120350 |
| 24 | picato.mp. | 19 |
| 25 | diclofenac.mp. or exp DICLOFENAC/ | 11804 |
| 26 | solaraze.mp. | 21 |
| 27 | solacutan.mp. [mp=title, abstract, original title, name of substance word, subject heading word, floating sub-heading word, keyword heading word, protocol supplementary concept word, rare disease supplementary concept word, unique identifier, synonyms] | 0 |
| 28 | photodynamic therapy.mp. or exp Photochemotherapy/ | 22013 |
| 29 | exp Aminolevulinic Acid/ or aminolevulinate.mp. or exp Photosensitizing Agents/ | 32250 |
| 30 | methyl aminolevulinic acid.mp. | 23 |
| 31 | MAL.mp. | 8756 |
| 32 | ALA.mp. | 41723 |
| 33 | BF-200 ALA.mp. | 18 |
| 34 | Ameluz.mp. | 5 |
| 35 | Alacare.mp. | 2 |
| 36 | Metvix.mp. | 64 |
| 37 | Luxerm.mp. [mp=title, abstract, original title, name of substance word, subject heading word, floating sub-heading word, keyword heading word, protocol supplementary concept word, rare disease supplementary concept word, unique identifier, synonyms] | 0 |
| 38 | Randomized Controlled Trials as Topic/ or RCT.mp. | 129373 |
| 39 | randomized controlled trial.mp. or exp Randomized Controlled Trial/ | 484460 |
| 40 | 38 or 39 | 599011 |
| 41 | skin surgery.mp. or exp Dermatologic Surgical Procedures/ | 48720 |
| 42 | 8 or 9 or 10 or 11 or 12 or 13 or 14 or 15 or 16 or 17 or 18 or 19 or 20 or 21 or 22 or 23 or 24 or 25 or 26 or 27 or 28 or 29 or 30 or 31 or 32 or 33 or 34 or 35 or 36 or 37 or 41 | 1030121 |
| 43 | 7 and 40 and 42 | 492 |
| 44 | limit 43 to (english language and humans) | 472 |
| 45 | limit 44 to (clinical trial, phase iii or clinical trial, phase iv or clinical trial or controlled clinical trial or randomized controlled trial) | 383 |

(b) Search in EMBASE via Ovid (June 13, 2018)

| **#** | **Searches** | **Results** |
| --- | --- | --- |
| 1 | actinic keratosis.mp. or actinic keratosis/ | 6512 |
| 2 | solar keratosis.mp. | 251 |
| 3 | senile keratosis.mp. | 19 |
| 4 | field change.mp. | 304 |
| 5 | actinically damaged field.mp. | 4 |
| 6 | field-cancerized.mp. or exp precancer/ | 16162 |
| 7 | 1 or 2 or 3 or 4 or 5 or 6 | 22604 |
| 8 | excision.mp. or exp excision/ or exp wide excision/ or exp local excision/ | 159216 |
| 9 | exp skin biopsy/ or shave.mp. | 62807 |
| 10 | curettage.mp. | 15152 |
| 11 | laser.mp. or exp carbon dioxide laser/ or neodymium YAG laser/ or exp erbium YAG laser/ or exp excimer laser/ or laser/ | 289158 |
| 12 | cryotherapy.mp. or exp cryotherapy/ | 26712 |
| 13 | exp cryosurgery/ or cryosurgery.mp. | 6113 |
| 14 | cryopeel*.mp. or exp skin surgery/ | 79390 |
| 15 | exp fluorouracil/ or exp fluorouracil plus salicylic acid/ or exp fluorouracil derivative/ or fluorouracil.mp. | 115652 |
| 16 | efudix.mp. | 197 |
| 17 | actikerall.mp. | 16 |
| 18 | 5-FU.mp. | 23887 |
| 19 | exp imiquimod/ or imiquimod.mp. | 8052 |
| 20 | aldara.mp. | 1071 |
| 21 | zyclara.mp. | 41 |
| 22 | ingenol mebutate.mp. or exp ingenol mebutate/ | 583 |
| 23 | picato.mp. | 100 |
| 24 | diclofenac.mp. or exp diclofenac/ or exp diclofenac derivative/ | 35983 |
| 25 | solaraze.mp. | 162 |
| 26 | solacutan.mp. [mp=title, abstract, heading word, drug trade name, original title, device manufacturer, drug manufacturer, device trade name, keyword, floating subheading word, candidate term word] | 0 |
| 27 | photodynamic therapy.mp. or exp photodynamic therapy/ | 26570 |
| 28 | exp phototherapy/ | 70060 |
| 29 | aminolevulinate.mp. or exp aminolevulinic acid/ | 9014 |
| 30 | exp aminolevulinic acid methyl ester/ or methyl aminolevulinic acid.mp. | 1303 |
| 31 | MAL.mp. | 16171 |
| 32 | exp nanoemulsion/ or exp photosensitizing agent/ or BF-200 ALA.mp. | 42793 |
| 33 | Ameluz.mp. | 36 |
| 34 | Alacare.mp. | 8 |
| 35 | Metvix.mp. | 590 |
| 36 | Luxerm.mp. [mp=title, abstract, heading word, drug trade name, original title, device manufacturer, drug manufacturer, device trade name, keyword, floating subheading word, candidate term word] | 0 |
| 37 | exp "randomized controlled trial (topic)"/ or RCT.mp. | 169956 |
| 38 | trial.mp. or exp "clinical trial (topic)"/ or exp "controlled clinical trial (topic)"/ or exp "phase 3 clinical trial (topic)"/ or exp "randomized controlled trial (topic)"/ or exp controlled clinical trial/ | 1813700 |
| 39 | 37 or 38 | 1820664 |
| 40 | exp curettage/ | 10967 |
| 41 | ALA.mp. | 36979 |
| 42 | 8 or 9 or 10 or 11 or 12 or 13 or 14 or 15 or 16 or 17 or 18 or 19 or 20 or 21 or 22 or 23 or 24 or 25 or 26 or 27 or 28 or 29 or 30 or 31 or 32 or 33 or 34 or 35 or 36 or 40 or 41 | 856282 |
| 43 | 37 or 38 | 1820664 |
| 44 | 7 and 42 and 43 | 1244 |
| 45 | limit 44 to (human and english language) | 1178 |
| 46 | limit 45 to (clinical trial or randomized controlled trial or controlled clinical trial or phase 3 clinical trial or phase 4 clinical trial) | 786 |

(c) Search in CENTRAL (June 13, 2016)

ID Search

#1 MeSH descriptor: [Keratosis, Actinic] explode all trees 331

#2 actinic keratos* 745

#3 solar keratos* 44

#4 senile keratos* 4

#5 field cancerization 50

#6 randomized controlled trial 705858

#7 precancerous lesion 193

#8 #1 or #2 or #3 or #4 or #5 or #7 943

#9 #8 and #6 563
